# Accuracy of dental implant positioning by dynamic or static computer-assisted implant surgery: a randomized controlled clinical trial

**DOI:** 10.1038/s41598-026-45931-1

**Published:** 2026-03-31

**Authors:** Karin Christine Huth, Jiří Hrkal, Michal Čičmanec, Frank Berlinghoff

**Affiliations:** 1 Department of Conservative Dentistry, Periodontology and Digital Dentistry, LMU University Hospital, LMU Munich, Goethestrasse 70, 80336 Munich, Germany; 2 Center for Stomatology, Slánská 1525, Kladno, 272 01 Czech Republic

**Keywords:** Computer-assisted implant surgery, Dynamic computer-assisted implant surgery, Static computer-assisted implant surgery, Dental implantology, Surgical guides, Randomized controlled trial, Implant planning, CBCT, Anatomy, Engineering, Health care, Medical research

## Abstract

While virtual planning of implant placement is common, reliable clinical transfer remains critical. This randomized clinical trial compared transfer accuracy between a miniaturized dynamic computer-assisted implant surgery system (dCAIS; DENACAM) and static computer-assisted implant surgery (sCAIS; BEGO). In 45 patients, 70 implants were placed (*n* = 35 per method). Deviations between planned and achieved positions were assessed by CBCT superimposition. Accuracy differences were analyzed using linear mixed-effects models (Type III F-tests; F, p-value, ICC) for 3D angular, 3D linear (implant base, tip), 2D mesio-distal, vestibulo-oral, and apico-coronal deviations. Ergonomic perception and covariate effects (age, sex, jaw, implant region, gap type) were analyzed using mixed-effects regression (β, 95% CI; α = 0.05). No significant accuracy differences were observed (*p* > 0.05). ICC values (0.00-0.56) indicated substantial patient-level variance. Mean 3D deviations (dynamic vs. static) were 4.89° ± 2.17 vs. 5.01° ± 2.13 (angular), 1.81 mm ± 0.69 vs. 1.55 mm ± 0.47 (base), 2.01 mm ± 0.72 vs. 1.78 mm ± 0.52 (tip). Ergonomic ratings were comparable. Covariate effects were limited to jaw (2D base deviation) and age (3D angular deviation) (*p* = 0.022, 0.015). This dCAIS may be equally suitable for implant plan transfer as sCAIS.

**Supplementary Information:**

The online version contains supplementary material available at 10.1038/s41598-026-45931-1.

## Background

Accurate three-dimensional implant positioning in relation to adjacent teeth and anatomical structures remains a key determinant of long-term implant success, as demonstrated in clinical studies^1–3^. Prosthetically driven implant planning based on cone beam computed tomography (CBCT) and virtual modeling is now standard practice and has improved predictability in implant dentistry. Virtually planned implant positions can be transferred clinically to the surgical site using computer-assisted implant surgery (CAIS), either by a static, template-based approach (sCAIS)^4,5^ or by a dynamic, real-time approach (dCAIS), or they may serve as a visual reference for a non-guided approach.

Depending on the clinical situation, sCAIS surgical guides may be tooth-, mucosa-, or bone-supported and can be designed as fully guided, semi-guided, or pilot-guided systems^6^. Systematic reviews and meta-analyses have demonstrated that sCAIS enables a high and predictable accuracy of implant placement compared with the non-guided approach^2^. Tissue support has been shown to influence accuracy, with tooth-supported guides generally achieving more favorable results than mucosa- or bone-supported designs^6,7^. A more recent meta-analysis reported the highest accuracy for unilateral and bilateral tooth-supported guides^8^. Given its well-established clinical use and documented accuracy, sCAIS represents an appropriate reference standard within a digitally assisted framework when assessing the performance of dynamic approaches;

dCAIS enables real-time intraoperative guidance by tracking the spatial relationship between the surgical instruments and the patient anatomy relative to the preoperative three-dimensional plan. In contrast to static guides, the drilling trajectory is continuously visualized during surgery, allowing the operator to adjust the drilling direction while remaining informed about the planned implant position^9–11^. Current systems implement this concept using different optical tracking technologies that register the position of the surgical handpiece and a patient reference within the coordinate system of the preoperative imaging dataset.

Compared with non-guided implant placement, both static and dynamic computer-assisted implant surgery have been shown to significantly improve the accuracy of transferring the virtual implant plan to the final implant position^12,13^. Several clinical studies have reported comparable accuracy between dCAIS and sCAIS^14–16^. A recent meta-analysis based on nine clinical studies concluded that dCAIS has achieved significantly higher placement accuracy than static surgical guides in recent years^17^. In a large randomized clinical trial including 120 patients, the highest accuracy was observed when static and dynamic approaches were combined^18^.

Beyond accuracy, dCAIS provides real-time intraoperative feedback during drilling^15^. This enables intraoperative flexibility and adjustment of the drilling trajectory if required, while maintaining visual guidance^19^. From a clinical perspective, dynamic approaches may facilitate implant placement in challenging situations, such as the molar region or in patients with limited mouth opening, and allow continuous access to relevant anatomical and planning information throughout the procedure^20^.

**Fig. 1:**
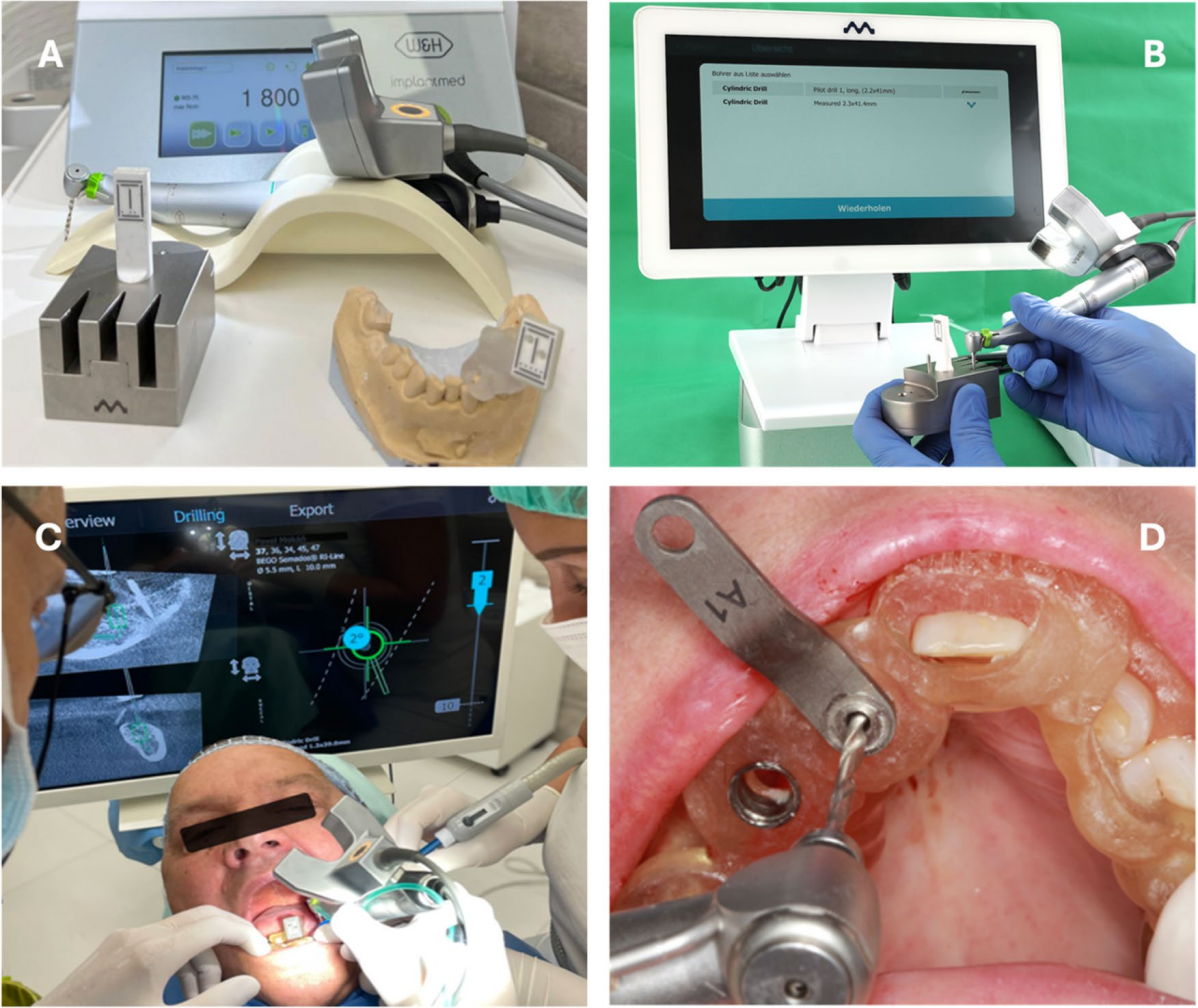
**A** Overview of the DENACAM system illustrating the stereoscopic camera mounted on the surgical motor, the metal drill calibration block with laser-engraved marker pattern, and the standardized ceramic reference marker positioned on a stone model using a patient-specific tray. **B** Drill registration using the metal calibration block and stereoscopic camera, followed by selection of the corresponding drill type from the DENACAM drill library displayed on the monitor. **C** Clinical application of the DENACAM system during guided drilling at site 37. The monitor displays sagittal and horizontal CBCT sections with the planned drilling trajectory. The green crosshair on the right marks the planned entry point, the filled blue disc (“2°”) indicates the current angular deviation (0° when aligned), the grey rings (“1, 2”) represent linear distance indicators, and the far-right scale shows current versus target drilling depth (bold, grey reference line). **D** Clinical situation using the BEGO surgical guide with metallic drill key (here A1) to adapt the guide sleeve diameter to the respective drill size.

Within the spectrum of dCAIS, substantial differences exist with regard to system size, tracking concepts, and clinical handling. Conventional dynamic systems typically rely on external multi-camera setups to track both a patient reference and the surgical instrument. In contrast, the DENACAM system (mininavident AG, Liestal, Switzerland) represents a miniaturized dCAIS concept based on a stereoscopic camera mounted directly on the surgical handpiece (Fig. 1A–C and D illustrates a representative clinical situation using a static surgical guide).

This camera-on-handpiece design allows unrestricted intraoperative mobility compared with space-demanding external camera systems. As reported previously^15,21^, the DENACAM system uses a marker-based registration concept in which a standardized intraoral reference marker is optically tracked intraoperatively by the stereoscopic camera mounted on the surgical handpiece. During treatment planning, the marker is positioned virtually within the CBCT coordinate system to establish the spatial relationship between the radiological dataset and the optical tracking system. In clinical practice, the marker position is selected by the operator to ensure an unobstructed line of sight for the camera, close proximity to the drilling site, and sufficient freedom of movement for the surgical handpiece. A patient-specific tray is subsequently fabricated to allow stable intraoperative positioning of the marker according to the planned location. In contrast to conventional dCAIS systems, continuous optical tracking of the drill itself during surgery is not required. Instead, drill geometry is calibrated prior to drilling, while only the patient position is tracked intraoperatively. This dedicated tracking concept reduces system complexity while maintaining real-time visualization of deviations between the planned implant position and the actual drilling trajectory. Accordingly, the present randomized clinical trial does not aim to re-evaluate static versus dynamic CAIS in general, but to assess the clinical accuracy and usability of this miniaturized, camera-on-handpiece dCAIS concept compared with an established sCAIS reference approach.

The primary objective of this randomized clinical trial was to compare the accuracy of implant plan transfer between this miniaturized dCAIS (DENACAM) and sCAIS (BEGO surgical guides). The primary hypothesis was that no differences would be observed in three-dimensional angular deviation or three-dimensional linear deviations at the implant base and tip. Secondary objectives were to evaluate two-dimensional linear deviations, including mesio-distal, vestibulo-oral, and apico-coronal deviations, as well as ergonomic perception and the influence of patient- and case-related factors (age, sex, jaw, implant region, gap type) on accuracy. The secondary hypothesis was that no significant effects would be present.

## Methods

### Patient and public involvement

There was no patient or public involvement in the trial’s design or reporting. The operator and study nurse conducted patient procedures; per the ethics application, the principal investigator led initiation and study design and, with the data manager, performed the data analysis.

### Trial design and registration

This randomized controlled clinical trial was conducted as a parallel group, one-center study (allocation ratio 1:1; unit of randomization: individual participant). The study was registered in the DRKS (DRKS-ID: DRKS00038401; registration date: November 18, 2025; URL: https://www.drks.de/search/de/trial/DRKS00038401/details) and was reported according to the CONSORT statement^22^.

### Trial setting

All implant treatments were performed at the Center for Stomatology, Kladno, Czech Republic between July 2019 and March 2023. Treatments were carried out by a single experienced operator with more than 20 years of clinical experience in dental implantology. Prior to patient recruitment, the operator completed a predefined familiarization phase comprising 20 implant drillings using the dCAIS, whereas sCAIS represented the routine standard technique. Study design and data evaluation were conducted at the Department of Conservative Dentistry, Periodontology and digital Dentistry, University Hospital, LMU Munich, Germany. The study was approved by the responsible ethics committees in Kladno, Czech Republic (approval date: July 8, 2019), and at the Medical Faculty of LMU Munich (No. 19–155), where the trial protocol and statistical analysis plan can be accessed.

### Eligibility criteria

All newly admitted patients at the Kladno center during the study period were screened for an indication for the insertion of at least one dental implant. Only patients for whom a CBCT scan was indicated based on the S2k guidelines on indications for 3D radiographic diagnostics and CAI^23^ were included. Patients were informed about the study both verbally and in writing, and written informed consent was obtained. Minors and individuals with disabilities who could not legally provide informed consent were excluded. Additional exclusion criteria were aligned with the above-mentioned guideline^23^.

### Intervention and comparator

#### Overview of workflow

**Fig. 2:**
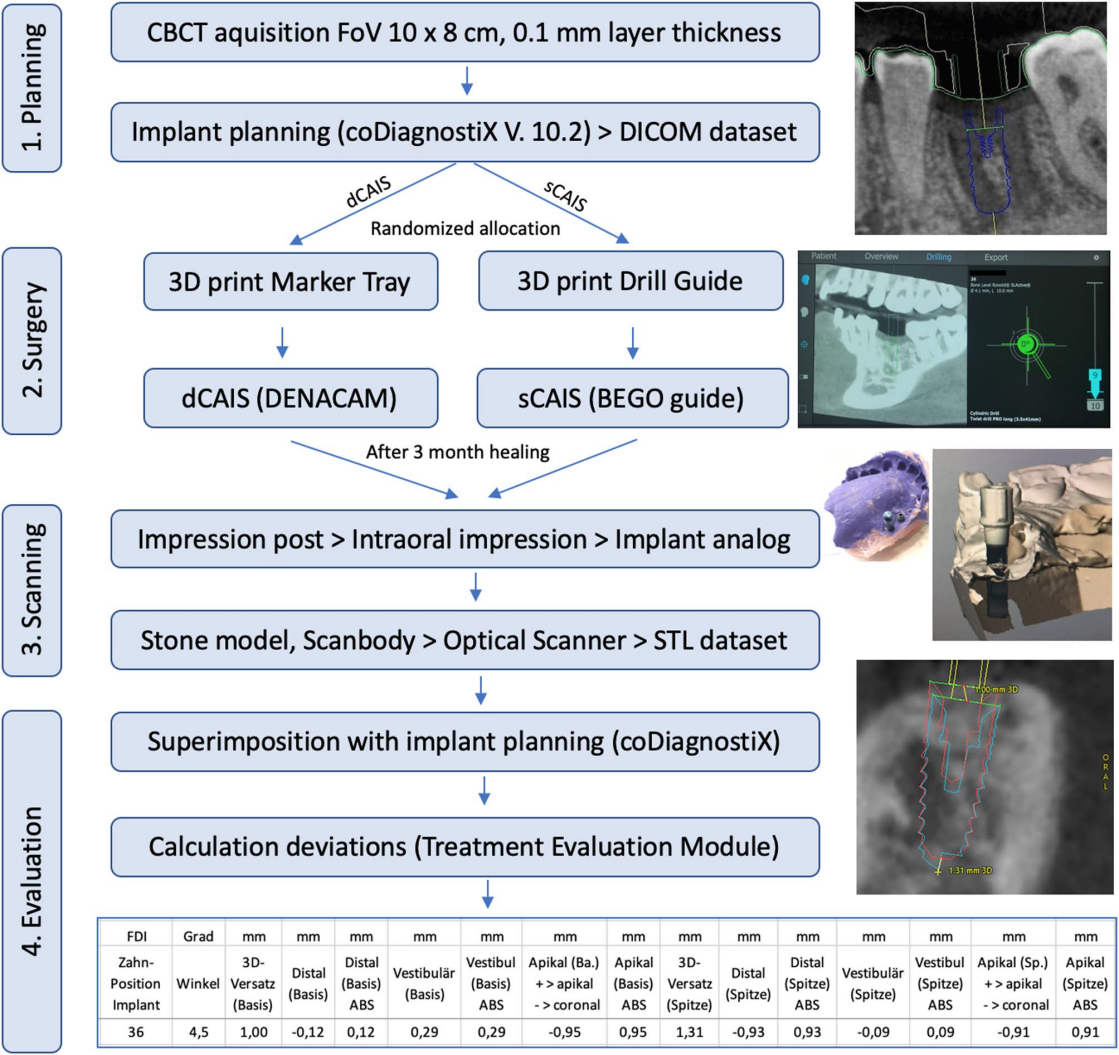
Methodology for measurement of 3D and 2D outcomes (1) Planning: Virtual implant planning (backward planning) in coDiagnostiX, based on CBCT data (DICOM dataset). Patients were randomized to dCAIS (DENACAM) or sCAIS (BEGO surgical guides). (2) Surgery: An STL dataset was generated by segmentation of the CBCT dataset and transferred for 3D printing of the DENACAM marker tray (after virtual planning of marker position) or surgical guide. Execution of CAIS according to drilling protocol. (3) Scanning: After 3 months of healing, an impression post was fixed to the implant, followed by impression taking, fabrication of a stone model with implant analog, and insertion of a scanbody for 3D optical scanning (given is an exemplary scanning image). (4) Evaluation: Superimposition of the scan STL dataset with the original planning data in coDiagnostiX; Calculation of 3D and 2D position/angulation deviations of final implant position compared to planning using the Treatment Evaluation Module (coDiagnostiX).

The complete workflow from preoperative planning to accuracy evaluation is illustrated in Fig. 2 and comprised four consecutive steps: (1) CBCT-based implant planning and randomization, (2) guided implant surgery using either dCAIS or sCAIS, (3) postoperative model-based scanning, and (4) three-dimensional accuracy evaluation.

#### CBCT acquisition and implant planning (common to both groups)

All patients underwent preoperative CBCT covering the planned implant site, adjacent anatomical structures, and the area required for fixation of either the surgical guide or the DENACAM marker tray (Fig. 2, Step 1). CBCT scans were acquired using the NewTom GO system (NewTom, Cefla Group, Imola, Italy) at 90 kV (pulse mode), 9.6 s exposure time, field of view 10 × 8 cm, and a voxel size of 100 μm.

CBCT data (DICOM format) were imported into implant planning software (coDiagnostiX 10.2 Producer, Dental Wings GmbH, Straumann Group). A prosthetically driven backward planning approach was applied. First, the desired prosthetic outcome (single crown or bridge) was defined, followed by virtual planning of implant position, number, length, and diameter.

For both groups, an STL dataset required for either fabrication of the surgical guide (sCAIS) or 3D printing of the DENACAM marker tray (dCAIS) was generated by segmentation of the CBCT dataset. No additional surface scan was performed.

Following implant planning, patients were randomized to either dCAIS (DENACAM) or sCAIS (BEGO) **(**Fig. 2, Step 1).

#### Guided implant surgery

##### Surgical procedure (common steps)

In both groups, implant placement was performed under local anesthesia using the same surgical motor (W&H Implantmed, W&H Dentalwerk, Bürmoos, Austria), contra-angle handpiece (WS 75 L 1:20), and the BEGO Guide surgical instrument set. A crestal midline incision was made to expose the surgical site in all cases.

Semi-guided drilling was performed according to the manufacturer’s protocol for BEGO Semados RI implants (diameter 3.75–4.1 mm; length 8.5–13 mm) using the following sequence: (a) center drill, (b) pilot drill (1.6 mm), (c) depth drill 2.5 mm, (d) depth drill 3.25 mm, and (e) optional non-navigated screw tap for implants with diameters of 3.75–4.1 mm. Implant insertion was not navigated in either group.

##### Group 1: dCAIS (DENACAM)

For dCAIS, a standardized ceramic reference marker (10 × 15 × 2 mm) was virtually positioned within the CBCT coordinate system (Fig. 2, Step 2). Marker location within the jaw (buccal or oral; ipsilateral or contralateral to the implant site) was selected by the operator to optimize line of sight and minimize the distance between marker and drill during surgery^24,25^.

A patient-specific STL-based marker holder was 3D printed and fixed to adjacent or antagonistic teeth to reproducibly position the marker intraoperatively. The DENACAM stereoscopic camera, mounted directly on the surgical handpiece, optically tracked the marker to register the patient position. Drill geometry was calibrated prior to drilling using a dedicated registration block (Fig. 1B).

All planning data, including CBCT, implant plan, and marker position, were exported from coDiagnostiX and transferred to the DENACAM system, which calculated the drilling pathway for the specific case. During drilling, the real-time position and angulation of the drill relative to the planned implant axis and depth were visualized on a monitor. Figure 1C shows a clinical situation navigating the drillhole for implantation at site 37. The monitor was positioned close to the surgical field to minimize eye movements between screen and operative site. The stereoscopic camera required an uninterrupted line of sight to the marker and could be adjusted in 15° increments for ergonomic positioning. Whenever the drill or camera position changed, the drill was re-registered using the sterilizable metal registration block to ensure correct drill identification by the system as shown in Fig. 1B. For description of the target screen please see Fig. 1C.

##### Group 2: sCAIS (BEGO)

For sCAIS, implant planning was identical to the dCAIS group. Planning data were exported, and a BEGO surgical guide with integrated sleeves was manufactured by the manufacturer (Fig. 2, Step 2). The guides were tooth-supported in dentate cases. In fully edentulous cases, guide fixation was achieved using temporary implants (Temporary ProImplant, diameter 2.1 mm, length 10 mm; LASAK s.r.o., Prague, Czech Republic). Correct seating was verified using inspection windows prior to drilling.

Semi-guided drilling followed the same drilling sequence as in the dCAIS group using the surgical guide for spatial control.

After implant insertion (ratchet adapter, torque wrench; BEGO), a healing cap was placed, the mucosa was sutured for closed healing, and an immediate postoperative radiograph was taken.

#### Postoperative impression and optical scanning (common to both groups)

After a minimum healing period of three months, an impression post (BEGO 3.75 PS OTI) was screwed into the implant (Fig. 2, Step 3). An impression was taken using an individualized tray and addition silicone material (Impression Tray Resin LC, Henry Schein, Langen, Germany; Variotime easy Putty + flow, Heraeus Kulzer GmbH, Hanau, Germany). An implant analog PS IMPA (BEGO) was inserted, and a stone model was fabricated.

A scanbody (BS1400, Medentika 3.75, Medentika GmbH, Hügelsheim, Germany) was attached to the implant analog, and the stone model was digitized using an optical desktop scanner (Dental Wings 7 Series, Dental Wings GmbH). The resulting STL dataset represented the three-dimensional position and angulation of the implant.

#### Accuracy evaluation

The STL data of the scanned model were superimposed with the original implant planning data in coDiagnostiX (Fig. 2, Step 4). Deviations between planned and actual implant position were automatically calculated using the Treatment Evaluation Module (coDiagnostiX 10.2 Producer Software) and included three-dimensional angular and linear deviations as well as two-dimensional linear deviations at implant base and tip.

### Outcomes

The primary outcomes were three-dimensional angular deviation and three-dimensional linear deviations at the implant base and tip. Secondary outcomes comprised two-dimensional linear deviations at the implant base and tip in the mesio-distal, vestibulo-oral, and apico-coronal (depth) directions.

In addition to the CAIS method (dCAIS, sCAIS), independent variables included patient-related factors (age, sex) and clinical factors such as implant location, gap type, marker position, template stability, and surgeon-reported ergonomics. Ergonomic perception was assessed after each implant drilling using a single-item 11-point Likert-type scale (0 = very poor, 10 = excellent)^26,27^. This rating represented an ad hoc, operator-reported measure and was not based on a validated multi-item ergonomics instrument.

### Sample size

Sample size was calculated using *Power and Sample Size Calculation* software (version 3.1.6; Dupont & Plummer)^28^. Based on the best available comparative in-vitro data at the time of study planning, the safety-critical two-dimensional apico-coronal deviation at the implant tip was selected as the planning parameter (mean difference 0.45 mm; SD 0.64 mm)^15^. Assuming a two-sided α of 0.05, 80% power, and a 1:1 allocation ratio, 33 implant drilling observations per group were required.

### Randomisation and blinding

Patients were randomly assigned in a 1:1 ratio to dynamic computer-assisted implant surgery (dCAIS; DENACAM) or static computer-assisted implant surgery (sCAIS; BEGO) using simple randomization with concealed allocation. Randomization was performed by an independent dental nurse using identical opaque lots drawn from a closed container. No blocking or stratification was applied.

Randomization was conducted at the patient level, and all implants placed within a given patient were treated using the assigned method.

Due to the nature of the interventions, blinding of the surgeon and participants was not feasible. Outcome assessors were blinded: datasets were pseudonymized, group identifiers were removed, and accuracy analyses were performed on de-identified files without device-related information.

### Statistical methods

Data were analyzed using SPSS Statistics (version 24.0; IBM, Armonk, NY, USA). Baseline group differences in categorical variables were assessed using chi-square tests, and age was compared using the Mann–Whitney U test due to non-normal distribution (Kolmogorov-Smirnov-test *p* = 0.006). Ergonomic ratings (11-point Likert scale) were analyzed using a linear mixed-effects model with method as a fixed effect and patient as a random intercept to account for clustering of multiple implants per patient.

For accuracy analysis, absolute deviations were calculated for bidirectional measures (mesio-distal, vestibulo-oral, apico-coronal). All 9 measured accuracy parameters were normally distributed (Kolmogorov–Smirnov-test, *p* > 0.05). Homogeneity of variance was given in 6 out of 9 parameters (Levene test). Accuracy outcomes were summarized descriptively (mean ± SD, median, 95% CI) and visualized using boxplots. Group comparisons were performed using linear mixed-effects models with method as a fixed effect and patient as a random intercept. Model results are reported as Type III F-tests with corresponding degrees of freedom and p-values. The proportion of variance attributable to patient-level clustering was quantified using the intraclass correlation coefficient (ICC). For deviation parameters without relevant patient-level clustering (intraclass correlation coefficient ≈ 0), fixed-effects models without random intercepts were applied. To explore factors influencing implant placement accuracy beyond method, multivariable linear mixed-effects models were constructed including age, sex, jaw (maxilla/mandible), implant region (anterior/posterior), and gap type as fixed effects, with patient included as a random intercept. Model estimates are reported as regression coefficients with 95% confidence intervals. Statistical significance was set at α = 0.05.

## Results

### Participant flow

**Fig. 3:**
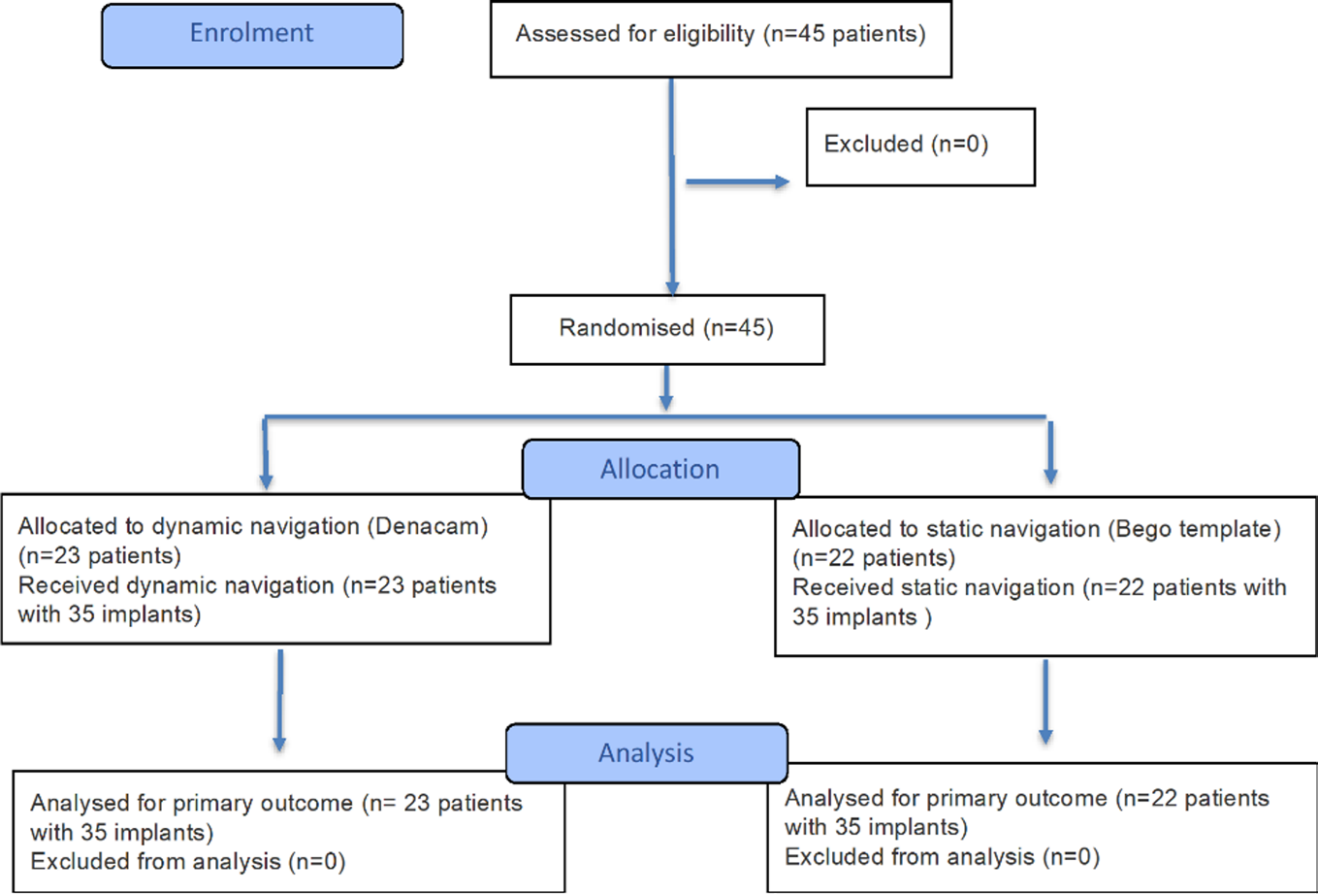
CONSORT flow diagram showing patient progress through the randomized trial (enrolment, allocation, follow-up, analysis) for both groups^22^.

The flow of participants through the trial is shown in Fig. 3. No participants were lost between allocation and analysis, and all randomized participants were included in the final analysis. Both the intervention and comparator were administered as planned.

### Baseline data

Forty-five patients (mean age 53.4 ± 12.8 years; range 22–82) received a total of 70 implants using computer-assisted implant surgery. Implant diameters ranged from 3.25 to 5.5 mm and lengths from 8.5 to 13 mm. Baseline demographic and clinical characteristics by method are summarized in Table 1. The two groups were comparable with respect to sex distribution, jaw, implant region, implant position, and type of edentulous space; patients treated with surgical guides were younger on average than those treated with DENACAM. In the DENACAM group, the ceramic marker was positioned predominantly on the buccal side and more frequently on the contralateral than the ipsilateral side. The fit of the surgical guides was good in all cases.

### Demographic and clinical characteristics for each group

**Table 1:** Demographic and clinical characteristics of the study cohort and placed implants by method. P-values indicate between-group comparisons of baseline characteristics (age: Mann–Whitney U test; all other variables: chi-square test) and are provided for descriptive purposes only. The significance level was set at 0.05.

	Sum	DENACAM	Surgical Guides	*p*-value
Patients (n)	45	23	22	0.36
Age (years)				0.01
Mean ± SD	53.4 ± 12.8	57.3 ± 13.1	49.3 ± 11.4	
Median (min - max)	52	57 (22–82)	47.5 (32–76)	
Gender				
Males	26	16	10	0.10
Females	19	7	12	
Implants (n)	70	35	35	
Jaw type				0.80
Maxilla (upper jaw)	23	11	12	
Mandible (lower jaw)	47	24	23	
Implant region				0.78
Anterior region	17	9	8	
Posterior region	53	26	27	
Implant position				0.74
Incisors	14	7	7	
Canines	5	4	1	
Premolars	21	12	9	
Molars	30	12	18	
Type of space				0.81
Single-tooth gap	47	23	24	
Free-end situation	16	9	7	
Fully edentulous jaw	7	3	4	
Marker position (dCAIS)				n.a.
Ipsilateral side		10	-	
Contralateral side		25	-	
Oral (lingual/palatal) side		5	-	
Buccal side		30	-	

### Outcome analysis

**Table 2:** Accuracy outcomes and comparison of dCAIS (DENACAM) and sCAIS (BEGO) for implant placement deviations.

Deviation parameter	DENACAM (*n* = 35) mean ± SD 95% CI median	Surgical guides (*n* = 35) mean ± SD 95% CI median	F (df1, df2)	*p*-value	ICC
3D angular deviation of the longitudinal implant axis (°)	4.89 ± 2.17 CI 4.14–5.64 4.90	5.01 ± 2.13 CI 4.28–5.74 4.7	0.002 (1, 41.31)	0.965	0.34
3D deviation of the implant base (mm)	1.81 ± 0.69 CI 1.58–2.05 1.79	1.55 ± 0.47 CI 1.39–1.71 1.44	1.80 (1, 45.30)	0.186	0.56
2D mesio-distal base deviation (mm)	0.56 ± 0.51 CI 0.38–0.73 0.42	0.54 ± 0.36 CI 0.42–0.67 0.55	0.031 (1, 44.51)	0.862	0.35
2D vestibulo-oral base deviation (mm)	0.52 ± 0.45 CI 0.37–0.67 0.40	0.53 ± 0.32 CI 0.42–0.65 0.53	0.023 (1, 68)	0.881	0.00
2D apico-coronal base deviation (mm)	1.44 ± 0.82 CI 1.16–1.72 1.42	1.20 ± 0.59 CI 1.00–1.41.00.41 1.22	0.986 (1, 41.60)	0.327	0.38
3D deviation of the implant tip (mm)	2.01 ± 0.72 CI 1.76–2.26 2.11	1.78 ± 0.52 CI 1.60–1.96 1.72	1.433 (1, 48.84)	0.237	0.33
2D mesio-distal tip deviation (mm)	0.95 ± 0.71 CI 0.70–1.19 0.81	0.86 ± 0.57 CI 0.66–1.05 0.81	0.413 (1, 51.66)	0.523	0.48
2D vestibulo-oral tip deviation (mm)	0.64 ± 0.41 CI 0.50–0.79 0.62	0.64 ± 0.54 CI 0.46–0.83 0.55	0.006 (1, 40.76)	0.939	0.15
2D apico-coronal tip deviation (mm)	1.39 ± 0.81 CI 1.11–1.67 1.36	1.16 ± 0.60 CI 0.96–1.37 1.13	0.887 (1, 42.77)	0.352	0.43

The first three columns present 3D and 2D deviations between digitally planned and clinically achieved implant positions by method (dCAIS, DENACAM; sCAIS, BEGO). Values are given in mm (angular deviation in degrees, °) as mean ± standard deviations SD, 95% confidence interval (CI), and median. The last three columns show the comparison of dCAIS and sCAIS across accuracy parameters using linear mixed-effects models with random intercepts for patients to account for clustering of multiple implants within patients. For parameters without relevant clustering at the patient level (ICC ~ 0), fixed-effects models were applied. F values and p values refer to Type III tests of the fixed effect “method”; df₁ and df₂ denote numerator and denominator degrees of freedom, respectively. ICC denotes the intraclass correlation coefficient.

**Fig. 4:**
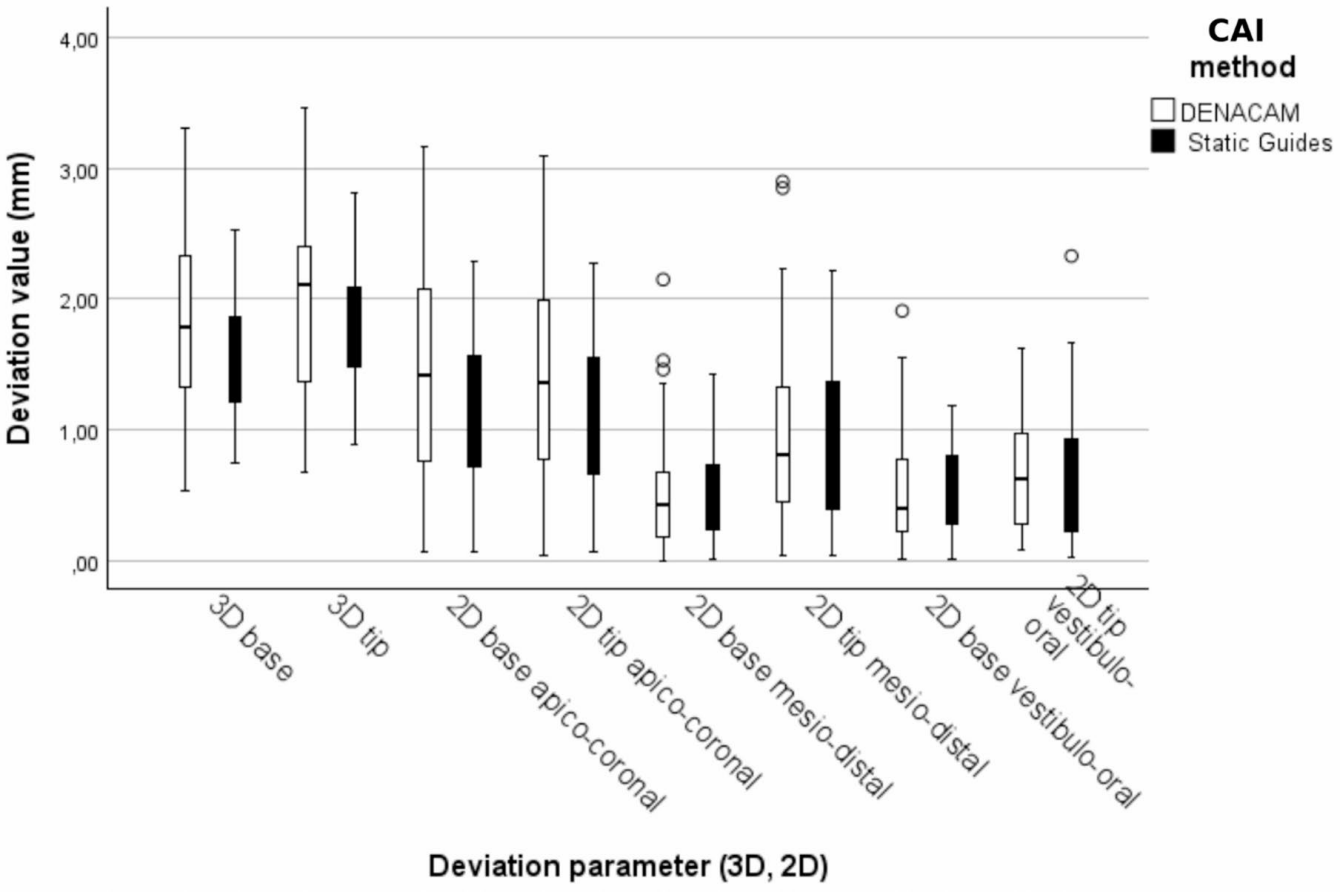
Clustered boxplots of 3D and 2D linear deviations (mm) between planned and achieved implant positions displayed for each deviation parameter with dCAIS (DENACAM) and sCAIS shown side-by-side. Boxes represent the interquartile range with median (line); whiskers indicate minimum and maximum value.

Table 2 (first three columns) and Fig. 4 summarize the three-dimensional and two-dimensional accuracy outcomes, defined as the deviations between the planned and the achieved implant positions, for each method. 

No significant differences between methods were observed for any of the investigated deviation parameters (Table 2, last three columns). Intraclass correlation coefficients indicated substantial clustering at the patient level for most outcomes, whereas no relevant clustering was observed for the two-dimensional vestibulo-oral base deviation. Overall, a considerable proportion of the variance was attributable to patient-level effects rather than the method. For the apico-coronal 2D deviation at the implant tip, which served as the planning parameter for sample size estimation, no significant difference between methods was observed (mean difference 0.23 mm; F = 0.887, *p* = 0.352).

Operator-rated ergonomics (0–10) were similar between the two methods (median = 8 in each group; interquartile range (IQR) for DENACAM = 7–8, for surgical guides = 7–9). Ergonomic ratings did not differ significantly between the two treatment methods (linear mixed-effects model with patient as a random effect; mean difference β = −0.12 points, 95% CI − 0.78 to 0.54; F(1, 53.3) = 0.13, *p* = 0.72). No harms or unintended effects were observed in either group.

### Ancillary analyses

Multivariable analyses showed no consistent associations for most examined covariates across the evaluated accuracy parameters (Supplementary Table S1). A significant association was observed for jaw location and three-dimensional deviation at the implant base, with higher deviations in the maxilla (β = 0.45 mm, 95% CI 0.07 to 0.84; *p* = 0.022; mean ± SD: 1.72 ± 0.53 mm vs. 1.66 ± 0.63 mm). Increasing age was associated with a slightly smaller angular deviation (β = −0.07° per year, 95% CI − 0.12 to − 0.01; *p* = 0.015); however, this finding should be interpreted with caution, as age was not a primary variable of interest and no causal inference can be drawn. No consistent effects were found for sex, region, gap type, or method.

### Multivariable mixed-effects models for implant placement accuracy

See Supplementary Table S1.

## Discussion

### Accuracy outcomes in context

In this RCT, dCAIS (DENACAM) was compared with sCAIS (BEGO surgical guides). Across all investigated accuracy parameters, including 3D angular deviation and 3D base/tip deviation as well as 2D mesio-distal, bucco-oral, and apico-coronal measures, no statistically significant differences were observed between methods. Accordingly, the primary hypothesis, that no differences would be observed between dCAIS and sCAIS in three-dimensional angular or linear deviations at the implant base and tip, was confirmed. The secondary hypothesis was partially rejected, as multivariable analysis identified associations between jaw and 3D base deviation and between age and angular deviation, while no differences were observed in two-dimensional deviations or ergonomic perception between methods. Mean 3D deviations ranged from approximately 1.5 to 2.1 mm, and mean angular deviations were around 4.9° in both groups, which is consistent with reports describing comparable accuracy across different clinical settings and levels of surgical experience^12,14^.

These findings are in line with recent RCTs and meta-analyses, reporting positional deviations of approximately 1–2 mm and angular deviations of around 4–5° for both sCAIS and dCAIS^12,29,30^. Earlier pooled observations suggested slightly lower errors for dCAIS (~ 1.1 mm entry, ~ 1.3 mm apex, ~ 3.6° angulation)^31^. However, such differences are commonly attributed to heterogeneity between in-vitro and clinical study designs, soft-tissue variability, and registration accuracy^31^. A recent systematic review and meta-analysis including 67 clinical studies reported entry deviations of 1.11 mm (95% CI, 1.02–1.19), apex deviations of 1.40 mm (95% CI, 1.31–1.49), and angular deviations of 3.51° (95% CI, 3.27–3.75), confirming that both dynamic and static CAIS routinely achieve clinically acceptable ≤ 2 mm accuracy^3^. Moreover, a subsequent RCT comparing dCAIS with oral-appliance–fixed patient trackers versus conventional guides for anterior implants found significantly better accuracy with dCAIS, entry 0.99 mm vs. 1.33 mm and angle 2.64° vs. 3.42° (*p* < 0.01), suggesting that tracker fixation method may influence performance^32^.

### System-specific aspects

The slightly higher deviations observed with the DENACAM system compared with conventional dCAIS systems may be related to system-specific technical characteristics. In DENACAM, the camera is directly attached to the surgical handpiece. The additional weight and off-axis positioning may introduce torque and dynamic imbalance potentially amplifying vibrations transmitted from the surgical motor during drilling. Such effects could potentially influence angular control, particularly during fine intra-operative corrections.

Moreover, this study evaluated the first clinical version of the DENACAM software, in which intra-operative orientation for angular correction was perceived as more demanding than in later versions, as reported in early clinical use. Operator-rated ergonomics did not differ significantly between DENACAM and BEGO surgical guides, indicating that ergonomics alone is unlikely to account for the observed differences. Taken together, while sCAIS demonstrated numerically higher accuracy in line with previous literature, the slightly higher deviations observed with DENACAM in this study are likely multifactorial, reflecting early-generation hardware-software integration rather than a fundamental limitation of the system.

### Clinical relevance

From a clinical perspective, the deviations observed in the present study fall within commonly accepted safety margins, typically requiring a buffer of approximately 2 mm to critical anatomical structures. When standard surgical protocols are followed, these deviations are therefore unlikely to compromise prosthetic outcomes or patient safety^6,8^. Overall, both CAIS methods demonstrated sufficient accuracy for reliable transfer of virtual implant plans to the surgical site.

Clinical decision-making regarding the choice of a CAIS approach should consider case complexity, anatomical constraints, and the knowledge of accuracy of the available techniques, including dCAIS, sCAIS, and non-guided approach. In cases with narrow safety margins or close proximity to critical anatomy, CAIS offering higher predictability may provide additional clinical confidence, whereas in less demanding situations alternative approaches may be equally appropriate.

### Modifiers of accuracy

In the DENACAM group, the reference marker was placed predominantly contralaterally and buccally to optimize line of sight and freedom of movement. Although marker positioning was left to operator preference, shorter marker–drill distances are known to improve stereoscopic triangulation accuracy. This observation is consistent with optical tracking data demonstrating reduced accuracy with increasing camera–workspace distance^24,25^.

Independent of the CAIS method, multivariable analysis indicated differences in positional accuracy between jaws, with higher accuracy at the implant base in the mandible compared with the maxilla, consistent with previous reports summarized in a recent meta-analysis^6^. A minor age-related association was observed for angular deviation only. As no corresponding effects were found for other accuracy parameters, this finding should be interpreted with caution.

### Workflow considerations

Although operator questionnaire ratings revealed no difference in perceived ergonomics between methods, static and dynamic CAIS differ in handling and intraoperative flexibility. Static surgical guides are simple and reproducible but inherently inflexible. In the present study, minor play of the guide sleeves was observed during drilling when a gap to the bone existed, whereas positioning sleeves close to the bone was occasionally limited in narrow interdental spaces.

With dCAIS (DENACAM), the monitor was positioned as close to the patient’s mouth as feasible to minimize visual shifts between the surgical field and the display. It allows real-time adjustment of angulation and depth, which may be advantageous in anatomically constrained or complex cases. In clinical practice, visual attention remained primarily on the surgical field, with brief glances to the screen for confirmation. Since completion of this study, the system has been further developed to include head-mounted displays (Straumann Falcon). However, CAIS information is not yet directly superimposed onto the clinical field.

## Limitations

Despite its prospective, controlled design and multivariable analysis, this study has limitations. The study was planned based on assumed group differences and variability derived from the in-vitro data available at the time of study design. In the clinical setting, the observed between-group differences, particularly for the safety-relevant apico-coronal deviation at the implant tip, were smaller; thus, while larger effects appear unlikely, small to moderate differences cannot be fully excluded. Further, all implants were placed by a single experienced surgeon with a uniform accuracy protocol. While this strengthens internal validity, it limits generalizability to less experienced operators, multi-surgeon settings, or alternative accuracy methods^14,33,34^. Implant position accuracy was assessed using an analog impression–cast–digitalization workflow rather than postoperative CBCT or direct intraoral scan. This multi-step approach may introduce cumulative measurement error due to impression material distortion, tolerances in implant analog seating, gypsum expansion during cast fabrication, and scanner/alignment-related uncertainty during extraoral digitization and superimposition. However, as the same standardized protocol and scanning workflow were applied consistently in both groups, any such measurement error is expected to affect both methods equally and is therefore unlikely to bias the comparative analysis. Moreover, the age effect on 3D angular deviation accuracy, should be interpreted cautiously, as potential age-related factors such as bone quality, anatomical access, or surgical complexity were not directly measured in this study. Finally, the findings are most applicable to routine implant therapy in partially edentulous patients under standard indications for guided surgery. Extrapolation to immediate implant placement, severely atrophic jaws, or markedly different clinical constraints should be made with caution.

## Conclusion

Both dCAIS using DENACAM and sCAIS using surgical guides demonstrated comparable and clinically acceptable accuracy for implant placement. The results support dCAIS as a reliable alternative to sCAIS, particularly when intraoperative flexibility is required. The choice of approach should ultimately be guided by patient-specific anatomy, clinical complexity, and operator preference. Future developments integrating hybrid workflows and advanced tracking technologies may further enhance precision and clinical usability.

## Supplementary Information

Below is the link to the electronic supplementary material.

Supplementary material 1 (DOCX 163.9 kb)

## Data Availability

The original data supporting the findings of this study is available from the corresponding author upon reasonable request.

## Abbreviations

CBCT: Cone Beam Computed Tomography
3D: Three-Dimensional
2D: Two-Dimensional
RCT: Randomized Clinical Trial
CI: Confidence Interval
SD: Standard Deviation
IQR: Interquartile Range
CAIS: Computer-Assisted Implant Surgery
sCAIS: Static Computer-Assisted Implant Surgery
dCAIS: Dynamic Computer-Assisted Implant Surgery
ICC: Intraclass Correlation Coefficient
