# Supplementary material for: Accuracy of dental implant positioning by dynamic or static computer-assisted implant surgery: a randomized controlled clinical trial
Source: Sci Rep. 2026 Mar 31;16:10997. doi: 10.1038/s41598-026-45931-1 (PMC13044292; doi:10.1038/s41598-026-45931-1)
Supplement: Supplementary file 1 — Supplementary material 1 (DOCX 163.9 kb) [file 41598_2026_45931_MOESM1_ESM.docx]

**
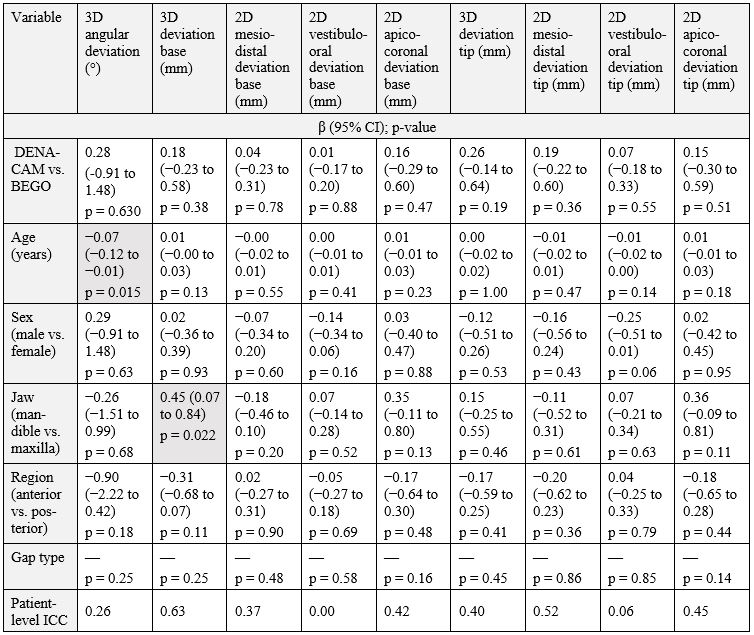
**

***Supplementary Table S1****: The table summarizes the results of the multivariable analysis across all deviation parameters. Columns show regression coefficients (β) with 95% confidence intervals (CI) and p-values for each deviation parameter. For categorical variables with more than two levels (gap type), p-values refer to the overall (Type III) effect; individual coefficients are not reported due to reference-category coding. Intraclass correlation coefficient (ICC) indicates the proportion of variance attributable to patient-level clustering.
All models were linear mixed-effects models including method, age, sex, jaw, implant region, and gap type as fixed effects, and patient as a random intercept. For outcomes with negligible or non-identifiable patient-level variance (ICC ≈ 0), fixed-effects models were applied (2D vestibulo-oral base deviation).*
